# Indoor Public Mask-Wearing Behavior Changes in Response to National, State, and Local COVID-19 Policies

**DOI:** 10.1097/PHH.0000000000001467

**Published:** 2021-12-21

**Authors:** Joshua R. Vest, Shama Cash-Goldwasser, Eleanor Peters Bergquist, Peter J. Embi, Virginia Caine, Paul K. Halverson

**Affiliations:** Department of Health Policy & Management, Indiana University Richard M. Fairbanks School of Public Health, Indianapolis, Indiana (Drs Vest and Halverson); Center for Biomedical Informatics, Regenstrief Institute, Indianapolis, Indiana (Drs Vest and Embi); Resolve to Save Lives, New York City, New York (Drs Cash-Goldwasser and Peters Bergquist); Marion County Public Health Department, Indianapolis, Indiana (Dr Caine); and Indiana University School of Medicine, Indianapolis, Indiana (Dr Embi).

**Keywords:** behavior observation techniques, COVID-19, mobile applications, public health

## Abstract

**Objective:**

To estimate changes in public mask-wearing behavior in response to public health policies during COVID-19.

**Design:**

Panel of observed public mask-wearing.

**Setting:**

Counts of adult behavior in Marion County, Indiana, between November 15, 2020, and May 31, 2021.

**Determinants of Interest:**

(1) Removal of state masking requirement; (2) introduction of the National Strategy for the COVID-19 Response and Pandemic Preparedness; (3) the Centers for Disease Control and Prevention (CDC) recommendation that vaccinated individuals did not need to wear masks in public; and (4) COVID-19 vaccine availability.

**Outcome:**

Percent observed with correct mask-wearing.

**Analyses:**

Fixed-effects models estimated the association between policies and mask-wearing.

**Results:**

Ending Indiana's mask requirement was not associated with changes in correct mask-wearing. The CDC's recommendation was associated with a decrease of 12.3 percentage points in correct mask-wearing (95% CI, −23.47 to −1.05; *P* = .032).

**Conclusions:**

Behavior encouraged by local mask requirements appeared to be resilient to changes in state policy. CDC recommendations appeared influential.

Policies requiring face masks in public have been one strategy to address the COVID-19 pandemic.^1^ Properly worn face masks are an inexpensive, accessible, and effective intervention to control the spread of SARS-CoV-2, which is the cause of COVID-19.^2^ Face masks have the dual benefit of limiting transmission by infectious persons and protecting the susceptible from exposure. Nevertheless, policies have varied between levels of government and over time.^3^ Moreover, masking was only one of multiple public health policy interventions instituted to address the pandemic.^4^ While commentaries and reports indicate these variations in public health policy created confusion among the public, their exact importance is not well known.^5^

When the next pandemic arises, public health policies will again be necessary to reduce transmission. Furthermore, public health agencies will be tasked with setting and enforcing policies within this complex dynamic.^3^ Insights into how public behavior changes in response to policy interventions may help guide such future response efforts. The objective of this study was to estimate changes in indoor public mask-wearing in response to 3 key public health policies that were implemented or changed during the pandemic: state mask requirements, federal mask policy, and vaccine availability.

## Methods

The association between COVID-19—related public health policies and mask-wearing behavior was assessed in a weekly, unbalanced panel of public behavior in a suburban/urban metropolitan area in Indiana over the course of 6 months. Trained observers collected counts of mask-wearing behavior. This study was approved by the Indiana University Institutional Review Board (2009738644A002).

### Setting and procedures

At each site, trained observers counted correct mask-wearing behavior of all observable individuals who (1) appeared to be older than 2 years^6^ and (2) whose faces were fully visible. During each observation session, a given individual was counted only once and mask status was recorded at the time of observation. Observers counted public behavior at 34 selected indoor sites across Marion County, Indiana, between November 15, 2020, and May 31, 2021. Mask status was recorded using a Web-based app (www.maskcount.com). Data were recorded independently for presumed males and for presumed females. The app automatically geocoded and time-stamped each observation session. In a training data set of images of mask-wearing behavior, the 17 trained observers exhibited very high agreement (κ = 0.92). This work is a longitudinal and policy-focused expansion of our prior cross-sectional study of mask-wearing behavior earlier in the pandemic.^7^

### Outcome variable

Correct mask-wearing was defined as any cloth face covering, N95, or surgical mask that covered the mouth and nose, including the nostrils, and extended below the chin.^6^

### Determinants of interest

The determinants of interests were the presence of those public health policies that could influence mask-wearing. Marion County and the state of Indiana required masks for 4 months prior to the start of the study. Indiana removed its requirement on April 6, 2021, but the county requirement remained. At the federal level, the National Strategy for the COVID-19 Response and Pandemic Preparedness (January 21, 2021) requested that Americans wear masks in public for 100 days.^8^ On May 13, 2021, the Centers for Disease Control and Prevention (CDC) recommended that vaccinated individuals did not need to wear masks in public.^9^ Indiana made COVID-19 vaccinations available in phases, with eligibility defined by profession and age. Over the study period, each of these state, federal, and vaccine availability policies were implemented or changed. Dummy indicators reflected the week these policies and vaccine availability were introduced.

### Covariates

Observation timing was described as morning, afternoon, or evening and weekday or weekend. We estimated crowd density by dividing the total number of individuals observed by the observation duration and into low, medium, and high tertiles. Weekly COVID-19 case counts and cumulative vaccination coverage were standardized by the resident population.

### Analyses

The unit of analyses was the data collection session (eg, a location on a specific date). Frequencies and percentages of the time-invariant and time-varying measures described the sessions. We organized the 907 sessions into a weekly longitudinal panel. The panel was unbalanced as each observation site was not observed each of the 29 weeks. Fixed-effects GLM fractional logistic regression models described the association between policy changes and the percentage of observed correct mask-wearing. Fractional logistic models are appropriate for continuous dependent variables, with values bounded between 0 and 1, which means the models are useful for proportion outcomes. Models were implemented in Stata 16, with location dummies accounting for the repeated measures, time dummies accounting for linear trends, the number of observations at each site as weights, and cluster robust standard errors. We created a fully adjusted model using all time-varying measures and excluding collinear measures and expressed coefficients as marginal effects. To illustrate correct mask-wearing over time, we graphed the weekly conditional means from the fully adjusted fractional logistic model.

### Supplemental data and analyses

In the final 10 weeks of the study, we recorded whether the observed sites had entrance signage about mask requirements, masks available for customers/patrons, hand sanitizer available, or a masked employee (ie, “greeter”) stationed at the entrance. We compared percentages between location types and changes in percentages after policy changes using χ^2^ tests.

## Results

The study period included a total of 907 observation sessions with 76 100 counts of mask-wearing behavior. Overall, the mean percentage of individuals correctly wearing masks indoors was 86% (Table 1). Correct mask-wearing was lowest at fitness locations (78%) and small retail stores (46%). Correct mask-wearing was more common among presumed females (87%) than presumed males (84%). As illustrated in the Figure, the percentage of individuals observed correctly wearing masks varied between 69% and 89% during the study period. Visual inspection of the weekly trends did not indicate any obvious changes in behavior.

**FIGURE:**
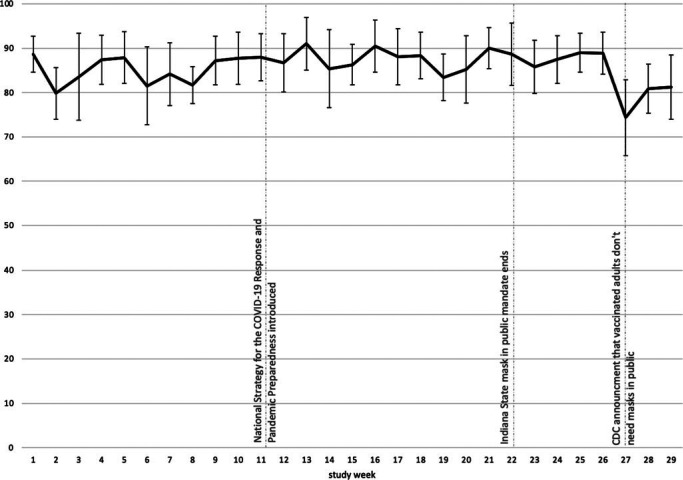
Percent Observed With Correct Mask-Wearing in Indoor Public Locations by Week, Marion County, Indiana Abbreviation: CDC, Centers for Disease Control and Prevention.

**TABLE 1:** Panel Characteristics and Percentage of Observed With Correct Mask-Wearing in Public Indoor Locations, Indianapolis, Indiana, From November, 15, 2020, to May 31, 2021

	n	Correct Mask-Wearing, % (SD)
Total	907^a^	85.8 (9.7)
Site, %		
Grocery and large retail	434	85.6 (7.5)
Shopping centers	148	84.4 (10.2)
Small retail stores	23	45.5 (28.8)
Civic and government	148	87.5 (11.1)
Fitness	70	77.8 (12.8)
Higher education	84	96.0 (3.4)
Time, %		
Morning (8 am-12 pm)	148	86.9 (9.6)
Afternoon (12 pm-6 pm)	604	85.7 (10.2)
Evening (after 6 pm)	155	85.3 (8.2)
Day, %		
Weekday	679	86.3 (10.0)
Weekend	228	84.7 (9.1)
Sex, %		
Male	453	84.4 (10.4)
Female	454	87.1 (9.0)
Crowd density,^b^ %		
Low	302	83.9 (14.0)
Medium	306	86.8 (7.6)
High	299	85.6 (9.7)

^a^In total, 76 100 observations across 907 sessions.

^b^Number of observations per minute.

In the fully adjusted model (Table 2), presumed females had nearly 3 percentage points higher for correct mask-wearing (marginal effect [ME] = 2.78; 95% CI, 1.97 to 3.59; *P* < .001). Correct mask-wearing was 2 percentage points lower in the evenings (ME = −2.12; 95% CI, −4.06 to −0.19; *P* = .03). Correct mask-wearing was nearly 3 percentage points higher at locations with highest crowd density (ME = 2.69; 95% CI, 0.46 to 4.93; *P* = .02).

**TABLE 2:** Marginal Effect of Site and Individual Characteristics on the Prevalence of Observed Correct Mask Mask-Wearing Behavior at Indoor Public Locations, Indianapolis, Indiana, From November 15, 2020, to May 31, 2021

	% Correctly Masked	
	Unadjusted Marginal Effect (95% CI)	Adjusted Marginal Effect (95% CI)
Time		
Before noon	Reference	Reference
Afternoon	−1.20 (−2.74 to 0.03)	−1.40 (−2.88 to 0.08)
Evening	−2.09 (−4.05 to −0.12)^a^	−2.12 (−4.06 to −0.19)^a^
Day		
Weekday	Reference	Reference
Weekend	−1.20 (−2.60 to 0.22)	−1.36 (−2.78 to 0.05)
Gender		
Male	Reference	Reference
Female	2.88 (2.12 to 3.65)^b^	2.78 (1.97 to 3.59)^b^
Density^c^		
Low	Reference	Reference
Medium	1.20 (−0.80 to 3.21)	1.67 (−0.50 to 3.83)
High	1.90 (−0.03 to 4.04)	2.69 (0.46 to 4.93)^a^
Prior week COVID-19 case rate^d^,^e^	1.26 (0.03 to 2.20)^f^	
Prior week cumulative COVID-19 vaccination^e^,^g^	−1.57 (−2.73 to −0.41)^f^	
Post–National Strategy for COVID-19 release	1.87 (−1.76 to 5.50)	1.87 (−2.15 to 5.60)
Post–CDC recommendations	−10.02 (−21.33 to 1.30)	−12.26 (−23.47 to −1.05)^a^
Post–Indiana State mask requirement	−4.12 (−14.19 to 5.95)	−3.00 (−11.90 to 5.90)
Vaccine available to general public (by age)^e^		
No general public	Reference	
80+ y	1.80 (−0.63 to 4.31)	
70+ y	1.66 (−0.14 to 3.47)	
65+ y	1.93 (−0.07 to 4.60)	
60+ y	2.73 (0.40 to 5.07)^a^	
55+ y	−10.72 (−12.21 to −9.22)^b^	
50+ y	−0.17 (−2.18 to 1.84)	
45+ y	3.21 (2.13 to 4.28)^b^	
40+ y	1.87 (−0.84 to 4.57)	
30+ y	6.22 (4.42 to 8.02)^b^	
16-12+ y	−0.62 (−3.25 to 2.02)	

Abbreviation: CDC, Centers for Disease Control and Prevention.

^a^*P* < .05.

^b^*P* < .001.

^c^Number of observations per minute.

^d^Per 1000 county population.

^e^Week vaccine made available to each age group, omitted from full model due to collinearity with weekly vaccinations and time dummies omitted.

^f^*P* < .01.

^g^Per 100 000 county population.

Neither the release of the National Strategy for the COVID-19 Response and Pandemic Preparedness in January nor the ending of Indiana's public mask requirement in April was associated with subsequent changes in correct indoor masking-wearing in the county (Table 2). As noted, Marion County still had a mask mandate in place during the entire study. The release of the CDC's guidance stating vaccinated individuals did not require masks in public was associated with a subsequent decrease of nearly 12 percentage points in correct mask-wearing (ME = −12.26; 95% CI, −23.47 to −1.05; *P* = .032) after controlling for other factors.

In unadjusted models, a 1-point increase in the prior week COVID-19 case rate (per 1000) was associated with a 1.3 percentage point increase in correct mask-wearing and a 1-point increase in the prior week's cumulative vaccination rate (per 100 000) was associated with 1.6 percentage point decrease in correct mask-wearing. Neither association persisted in the adjusted model.

### Supplemental analyses

In the last 10 weeks of the study, the majority of observed indoor locations had signs requiring masks (81%). Across sites, efforts to encourage mask-wearing were least common at fitness locations and smaller retail stores. Overall, the individual policies and procedures of observation sites did not change dramatically pursuant to changes in state and national policies (see the Appendix).

## Discussion

In this longitudinal panel of direct observation of public behavior, a majority of observed individuals correctly wore a face mask during the period when a county mask requirement was in place. While concerns exist about differing state and local public health policies,^3^ these findings suggest that public behavior may be robust to changes in state policy when a local requirement is in place. The continued prevalence of indoor mask-wearing may be due, at least partially, to a local policy that included financial penalties for individual and business violations throughout the study period. In contrast, Indiana's state policy was not enforced.^10^ Also, efforts such as signage to encourage mask-wearing were the norm at most observation sites, which may have further supported public behavior. These findings are similar to a prior national analysis that suggested that state-level mask requirement did not affect self-reported mask-wearing.^11^

Federal policy, in the form of a strategic plan, was not associated with behavior. January 2021's National Strategy for the COVID-19 Response and Pandemic Preparedness represented a stronger endorsement of mask-wearing in public than the prior administration.^12^ However, the change in overall federal policy about masking was not associated with observed mask-wearing behavior in this panel. The absence of change may be attributable to the limited scope of the federal policy: mask-wearing was *encouraged* for all and only required in federal buildings and during interstate and international travel.^8^ Moreover, the change in the tone of federal policy occurred during a time when masking was already relatively common and when both the state and county requirements were in place.

However, the CDC recommendation that fully vaccinated individuals did not need to be masked in public was associated with a significant reduction in indoor mask-wearing behavior. This finding is consistent with the 2020 increase in mask-wearing when the CDC *recommended masking* in public.^13^ While individuals may not be necessarily reacting to the science of recommendations, it is possible that excitement about having achieved a perceived pandemic milestone or the more intensive media coverage may have altered behavior.

### Limitations

While our fixed-effects approach has strengths, this study faces limitations in internal and external validity. Our study period did not include the introduction and ending of the county-level mandate, so we are unable to assess the impact of changes in local policy. Findings may not be generalizable to other geographies or other types of settings (eg, restaurants). We do not have a comparison group to account for other external events that may have occurred at the same time as key policy changes. For example, the CDC recommendation occurred the same week the state of Indiana announced availability of vaccinations to those 12 years and older. However, the increased availability of vaccine for more members of the public is likely not an alternative explanation as this age group did not begin to be fully vaccinated until nearly 3 weeks later (eg, the end of our study period).

## Conclusions

Over the course of the COVID-19 pandemic, health policies at all levels of government evolved and changed. However, mask policies, and in particular mask requirements in public, did not achieve universal mask adherence and variation existed across settings and by gender. Nevertheless, the majority behavior encouraged by local mask requirements appeared to be resilient to changes in state-level policy, illustrating the importance of local government and local public health agency actions in communicable disease prevention. CDC recommendations appeared to be influential.

**Implications for Policy & Practice**

- Public health policies, as in the case of face-masking, were not always consistent between levels of government, varied over time, and were only one concurrent intervention to address the pandemic.
- Local policies may still be influential even when not aligned with the state, particularly when the local policy is enforced more stringently.
- Federal strategic plans may not be very influential on individual behavior; however, CDC recommendations may still be important.

## Appendix

**APPENDIX:** Prevention Efforts at Entrance of Indoor Locations During Last 10 Weeks of the Study

	Signs, %	Mask Distributed, %	Hand Sanitizer, %	Masked Employee, %
Total	81.3	14.2	45.7	20.8
Site, %				
Grocery and large retail	91.4^a^	12.1^b^	27.6^a^	24.1^b^
Shopping centers	84.6	0.0	75.0	0.0
Small retail stores	37.5	0.0	0.0	14.3
Civic and government	100.0	25.0	75.0	58.3
Fitness	0.0	0.0	100.0	0.0
Higher education	100.0	62.5	75.0	0.0
Indiana State mask mandate				
Before	82.6	17.4	52.2	26.1
After	81.0	13.3	43.9	19.3
CDC guidelines on public mask-wearing				
Before	76.1	16.4	52.2	22.4
After	90.0	10.3	34.2	20.8

Abbreviation: CDC, Centers for Disease Control and Prevention.

^a^*P* < .001.

^b^*P* < .01.
