# Supplementary material for: Indoor Public Mask-Wearing Behavior Changes in Response to National, State, and Local COVID-19 Policies
Source: J Public Health Manag Pract. 2021 Dec 21;28(3):292–8. doi: 10.1097/PHH.0000000000001467 (PMC8963438; doi:10.1097/PHH.0000000000001467)
Supplement: Supplementary file 1 [file jpump-28-292-s001.docx]

| **Appendix 1: Facilitators and barriers to telehealth service provision at Health Department clinics in State 1 and State 2 (N=20 interviewees).^** |  |  |
| --- | --- | --- |
| **Facilitators of telehealth service provision** | **Representative Quotation** |  |
| **Policy/Structural Factors** |  |  |
| Electronic infrastructure and technology | “I guess the fact that the majority of the population has a cell phone and can communicate by phone.” (State 1) |  |
| Medicaid reimbursement policy | “It was an approval from Medicaid that gave us permission to offer telehealth to Medicaid patients.” (State 1) |  |
| **Organizational Factors** |  |  |
| Patient education to utilize telehealth | “Us having multiple handouts to give the patients, as far as education, that went well with our nurses. They would stand at the car and make sure that they understood and get their consent signed.” (State 1) |  |
| Clinician and staff training for telehealth | “It was having our clinicians educated and trained how to provide a new service outside the box of being in a physical building, one-on-one, face-to-face with the patient.” (State 1) |  |
| **Provider Buy-In** | “We just tried to make sure that all of our patients were covered so that they were not without birth control. We just did whatever was necessary during that time for both the patients and for us.” (State 1) |  |
|  | “Just the willingness of the agency and the providers and the nurse practitioners to be on board with providing services this way because if we didn’t have the backing of the agency and the nurse practitioners to call it in, we wouldn’t be able to provide it.” (State 2) |  |
| **Patient Acceptance** | “The patients also, accepting that and handling that very well, I think was the key success to doing the telehealth.” (State 1) |  |
| **COVID-19 Pandemic** | “The factor that actually initiated us providing telehealth was the pandemic and patients not feeling comfortable in coming into the clinic. That was the big factor that initiated the telehealth visit.” (State 1) “Definitely COVID-19 has facilitated it [telehealth]. We just had to figure out a way to not have a client in a room with a nurse for a long period of time.” (State 2) |  |
| **Barriers to telehealth service provision** |  | **Representative Quotation** |
| **Policy/Structural Factors** |  |  |
| Limited infrastructure and technology | “My understanding of telehealth means that you have to have both video and audio capability, which our technical, our IT services, we do not have that availability to be able to do that.” (State 2) |  |
| No policies or procedures for implementation | “We do not have telehealth capabilities at our department. We do not have the software, the equipment, no policies or procedures.” (State 2) |  |
| Telehealth billing for nurses | “My understanding is for our preventive health, our nurses cannot bill for telehealth services. That would be a barrier.” (State 1) “Actually, nursing staff are not allowed to, and this is per billing guidelines and nurse practice acts and things of that nature. They’re not allowed to provide telehealth services.” (State 2) |  |
| Inherent limitations to telehealth service provision | “The barriers would be you can’t insert an IUD through telehealth.” (State 1) “The barriers are just that when you’re providing telehealth, you’re not seeing the patient. That physical connection is not there, you can’t read body language.” (State 2) |  |
| **Organizational Factors** |  |  |
| Scheduling/staffing limitations | “At times when it does get a little bit backed up, at times trying to see the patients that have to come in and then calling the patients. That’s the nurse practitioner that has to be the one to call the patients and do the telemedicine visit, so working out schedules ensuring that the schedule is appropriate.” (State 1) “Just staffing availability because even for a telehealth service, I’m currently the only nurse at this site and have just now been released from COVID-19 response efforts. A limitation would have been staffing availability to even meet the telehealth need if we had been in the practice of doing so.” (State 2) |  |
| **Provider Comfort Level** | “Just because it was so new and so outside the box of what we were used to, I think people just becoming comfortable with doing that and not physically being able to see a patient when they’re talking to them…that was probably one of the biggest barriers.” (State 1) |  |
| **Patient Factors** | “I think sometimes when we call the patient they don’t recognize the number and maybe they don’t answer. People are wary about answering calls that they don’t know the number. Another challenge could be that maybe the patient just is unsure about all of this and if this is what they need to be doing.” (State 1) “…if you come into the clinic you can see visually, but you trying to explain to somebody over the phone with an interpreter, how you use some type of birth control or what you need to do or how you need to do it, sometimes is not as helpful. Some of our people can’t read or they can’t understand what somebody is saying.” (State 2) |  |
| **No barriers to telehealth service provision** | “We really haven’t had any barriers because it was pretty cut and dry. We knew what we needed to do. We knew what was required to be on our EHR note.” (State 1) |  |

^ Data are from Key-informant Interviews and quotes are representative of each topic for each state, where applicable.
